# Editorial for Special Issue “Molecular Studies of Female Pregnancy Disorders”

**DOI:** 10.3390/cimb47050337

**Published:** 2025-05-08

**Authors:** Alicia Rodríguez Barbero

**Affiliations:** 1 Departamento de Fisiología y Farmacología, Universidad de Salamanca, 37007 Salamanca, Spain; barberoa@usal.es; 2 Biomedical Research Institute of Salamanca (IBSAL), 37007 Salamanca, Spain

## 1. Introduction

Pregnancy is a complex biological process that requires precise molecular regulation to ensure maternal and fetal health. Disruptions to these mechanisms can lead to pregnancy disorders such as preeclampsia, gestational diabetes, recurrent pregnancy loss, and preterm birth, each of which poses significant risks to both mother and child. Despite advancements in maternal–fetal medicine, many underlying molecular pathways remain elusive, limiting the development of effective diagnostic, preventive, and therapeutic strategies.

This Special Issue, focusing on molecular studies of pregnancy disorders, presents a collection of pioneering research works that advance our understanding of the molecular foundations of these conditions. The articles included in this issue explore novel biomarkers, genetic and epigenetic factors, immunological pathways, and emerging therapeutic approaches, providing valuable insights into pregnancy complications at a molecular level.

Several studies featured in this Special Issue have expanded our knowledge of the genetic predispositions that contribute to pregnancy disorders. By identifying key genetic variants and epigenetic modifications associated with complications such as preeclampsia and gestational diabetes, these findings offer promising avenues for early detection and personalized interventions. Additionally, research into immune system dysregulation has shed light on its role in pregnancy loss and preterm birth, emphasizing the importance of immune balance in maintaining a healthy pregnancy.

While these contributions significantly enhance our molecular understanding, major knowledge gaps remain. The intricate crosstalk between maternal and fetal tissues, the influence of environmental and lifestyle factors on molecular pathways, and the integration of multi-omics data for comprehensive disease modeling warrant further exploration. Future research should focus on large-scale cohort studies, innovative in vitro and in vivo models, and the application of artificial intelligence to decipher complex molecular interactions underlying pregnancy disorders.

We extend our sincere appreciation to all contributing authors for their invaluable research, as well as to the peer reviewers whose expertise has enhanced the quality of this Special Issue. Their collective efforts have enriched the scientific discourse and will undoubtedly inspire further exploration in this critical field.

As we continue to address the challenges posed by pregnancy disorders, we hope that the advancements presented in this collection will serve as a foundation for future discoveries, ultimately leading to improved maternal and fetal outcomes. We encourage readers to engage with these contributions and to build upon the knowledge presented herein to drive innovation in maternal–fetal medicine.

## 2. An Overview of Published Articles

In our first contribution, Ho-Yeon [1] provides crucial insights into the molecular mechanisms by which cigarette smoking influences preeclampsia risk, specifically through the regulation of soluble FMS-like tyrosine kinase-1 (sFlt-1), vascular endothelial growth factor (VEGF), and endoglin (sEng-1). Using a controlled smoking mouse model, the researchers demonstrate that exposure to cigarette smoke significantly decreases serum sFlt-1 levels, a key antiangiogenic factor associated with preeclampsia. Notably, the aryl hydrocarbon receptor (AhR) appears to play a pivotal role in this process. When an AhR agonist was administered, sFlt-1 levels decreased, while the AhR antagonist reversed this effect, restoring sFlt-1 expression. These findings suggest that AhR activation may mediate the protective effects of smoking on preeclampsia by modulating sFlt-1 levels. While VEGF levels were altered by exposure to one cigarette, the effect was not consistent in the two-cigarette-exposed group, and placental expression patterns of sFlt-1, VEGF, and sEng were variable. Nevertheless, the study highlights AhR as a potential target for novel therapeutic strategies in preeclampsia treatment. Future research should explore AhR-modulating agents as a safer alternative to harness the protective molecular effects observed in smoking, without its harmful consequences.

Second, Olena et al. [2] focus on prenatal hypoxia as a significant risk factor for the development of severe cardiovascular diseases in both children and adults, though this remains an underexplored issue in pediatric cardiology. This study evaluates the cardioprotective effects of L-arginine, Thiotriazoline, Angioline, and Mildronate on the cardiovascular system of rats affected by prenatal hypoxia. The results indicate that Angioline demonstrated the most sustained cardioprotective effect, maintaining its benefits even a month after treatment discontinuation, with a notable reduction in ST2 nitrotyrosine, a marker of oxidative stress. Thiotriazoline and L-arginine exerted antioxidant effects and significantly increased eNOS expression and HSP70 concentration, suggesting their role in enhancing nitric oxide bioavailability and cellular protection. Meanwhile, Mildronate also increased eNOS expression and HSP70 concentration, but, unlike the other treatments, did not exhibit antioxidant properties or reduce nitrotyrosine levels. These findings highlight the potential of NO system modulators as therapeutic agents to counteract cardiovascular damage resulting from prenatal hypoxia. The varying mechanisms of action among these drugs suggest the possibility of tailored treatment strategies to address different aspects of hypoxia-induced cardiac dysfunction. Future research should focus on optimizing dosing regimens and exploring clinical applications for these cardioprotective agents.

The third contribution is that of Tossetta et al. [3], which investigates HtrA serine peptidase 1 (HTRA1), a secretory protein with serine-protease activity that plays a crucial role in cellular processes, and particularly in early placental development. This study aimed to define the functional role of HTRA1 in preeclampsia (PE) using in vitro models of the human placenta. The findings demonstrate that oxidative stress significantly increases HTRA1 expression in both BeWo and HTR8/SVneo cells, which serve as models for syncytiotrophoblast and cytotrophoblast cells, respectively. Furthermore, functional experiments revealed that HTRA1 is pivotal in cell motility and invasion. Specifically, HTRA1 overexpression enhanced cytotrophoblast motility and invasion in the HTR8/SVneo model, while HTRA1 silencing reduced these. These results suggest that HTRA1 is critical for extravillous cytotrophoblast invasion and motility during the early stages of placentation, processes that are known to be dysregulated in preeclampsia. The study highlights HTRA1 as a potential molecular player in the pathogenesis of PE, providing insights into its role in trophoblast function and proposing its use as a potential target for future therapeutic strategies. Further research is needed to explore its mechanistic pathways and clinical implications in pregnancy disorders.

The article by Popiel-Kopaczyk et al. [4] suggests that testin, a cytoskeletal-associated protein, is essential for maintaining cell–cell junction integrity and focal adhesion plaques, playing a role in cell motility and actin filament regulation. Alterations in testin expression have been linked to various malignancies, including cervical cancer, though its role remains incompletely understood. This study aimed to evaluate testin expression in cervical cancer tissues and to analyze its correlation with clinical data, the Ki-67 antigen (a proliferation marker), and the p16 protein (a marker of cell cycle dysregulation). The findings revealed that testin expression was significantly lower in cervical cancer cases compared to controls. Inverse correlations were observed between testin and Ki-67 expression as well as between testin and p16 expression, suggesting that testin downregulation is associated with increased cellular proliferation and dysregulated cell cycle control. Western blot analysis of cervical cancer cell lines confirmed these results, showing weaker testin expression, particularly in HPV-negative cell lines. These findings highlight testin as a potential biomarker in cervical cancer, with its expression patterns providing valuable diagnostic insight when analyzed alongside Ki-67 and p16. This work suggests that the combined assessment of these markers may enhance cervical cancer diagnostics, paving the way for improved stratification and potential therapeutic targeting in cervical malignancies.

The fifth paper featured in this Special Issue is a review by Fernandes et al. [5] on the hypertensive disorders of pregnancy (HDP), which pose significant risks to both maternal and fetal health. Emerging evidence suggests a strong link between testosterone levels and preeclampsia (PE), potentially mediated through androgen receptors (ARs). However, the underlying mechanisms of this remain unclear. This study provides a comprehensive review of research spanning two decades (1998–2022) to examine the association between testosterone and HDP, including a novel perspective from pregnancies in transgender men. The findings indicate that placental samples from PE cases show increased AR mRNA expression, while ex vivo studies suggest that testosterone impairs vasorelaxation, promoting hypertension. Epidemiological data further support the association between elevated testosterone levels and PE. Notably, studies involving transgender men—who undergo exogenous testosterone therapy—suggest that testosterone administration may increase the risk of PE without negatively impacting fetal development. These insights underscore testosterone as a potential contributor to PE pathophysiology, highlighting the need for further investigation into androgen-mediated vascular dysfunction in pregnancy. Understanding this relationship could pave the way for novel therapeutic approaches aimed at mitigating HDP risk while preserving maternal and fetal health.

Next, Tobaco’s review [6] focuses on future research into personalized approaches to prevention and treatment based on individual risk factors and molecular profiles. Preeclampsia incidence has significantly increased over the past 30 years, yet its etiology and molecular mechanisms remain poorly understood. Multiple factors contribute to its development, including altered angiogenesis, inflammation, maternal infections, obesity, metabolic disorders, gestational diabetes, and autoimmune diseases. Among the most promising areas of research, the imbalance of maternal angiogenic factors and its impact on vascular function has gained significant interest. Additionally, studies focusing on placental oxidative stress and maternal immune response have revealed intriguing findings, suggesting complex pathophysiological interactions. However, further research is required to determine the relative importance of each cause and assess the efficacy of preventive and therapeutic interventions aimed at reducing preeclampsia incidence. A deeper understanding of the distinct subtypes of preeclampsia and their specific pathophysiological mechanisms is crucial. Identifying early clinical markers and risk stratification tools could significantly improve early detection and management, ultimately reducing the burden of this life-threatening pregnancy disorder.

## 3. Conclusions

The contributions in this Special Issue provide valuable insights into the molecular and physiological mechanisms underlying pregnancy-related disorders, particularly preeclampsia (PE), prenatal hypoxia, and cervical cancer. These studies collectively highlight the significance of angiogenic factors, oxidative stress, hormone regulation, and cytoskeletal proteins in disease development and progression.

### 3.1. Molecular Mechanisms in Preeclampsia

Ho-Yeon’s study [1] demonstrates that cigarette smoke exposure alters sFlt-1 levels via AhR activation, offering a potential therapeutic target to harness the protective molecular effects observed in smokers without the associated health risks.

Further, Tossetta et al. [3] identify HTRA1 as a key regulator of trophoblast invasion and motility, further implicating oxidative stress in preeclampsia pathogenesis.

Meanwhile, Fernandes et al. [5] provide evidence linking elevated testosterone levels with PE development, highlighting androgen receptors (ARs) as potential mediators of hypertensive disorders in pregnancy.

### 3.2. Cardiovascular Consequences of Prenatal Hypoxia

Olena et al. [2] emphasize the long-term cardiovascular risks associated with prenatal hypoxia, demonstrating the cardioprotective effects of L-arginine, Thiotriazoline, Angioline, and Mildronate. Their study suggests that NO system modulators may help mitigate hypoxia-induced cardiac dysfunction with potential applications in pediatric cardiology.

### 3.3. Role of Testin in Cervical Cancer

Popiel-Kopaczyk et al. [4] investigate testin expression in cervical cancer, demonstrating an inverse correlation with Ki-67 and p16, suggesting its role as a potential biomarker for cancer progression and diagnostic stratification.

### 3.4. Future Directions in Pregnancy-Related Disorders

Tobaco’s review [6] underscores the importance of personalized approaches to preeclampsia prevention and treatment, emphasizing the need for early clinical markers and risk stratification tools to improve patient outcomes.

## 4. An Integrative Summary

Recent advances in molecular biology have significantly deepened our understanding of pregnancy disorders. These insights include mechanisms of placental development, vascular remodeling, immunoregulation, and metabolic adaptations, offering novel avenues for early diagnosis and intervention.

One of the central themes emerging from current research is the molecular basis of placental dysfunction, a common denominator in several pregnancy-related complications. Lin et al. [7] demonstrate that the dysregulation of the YAP signaling pathway impairs trophoblast function, leading to deficient placental invasion and, consequently, complications such as preeclampsia and fetal growth restriction. This discovery was complemented by findings from Rybak-Krzyszkowska et al. [8], who reviewed the molecular biomarkers of preeclampsia, including oxidative stress markers and antiangiogenic factors such as sFlt-1.

Vascular remodeling, a key physiological adaptation during pregnancy, has also been a focal point in this area of research. Stevenson et al. [9] highlighted how impaired signaling through pathways like VEGF and nitric oxide underlies disorders such as intrauterine growth restriction and preeclampsia, emphasizing the importance of endothelial function in gestational health. Likewise, the role of extracellular matrix proteins such as biglycan and decorin was explored by Halari et al. [10], who found that the dysregulation of these proteins contributes to abnormal placental architecture and inflammation via TLR and TGF-β signaling.

The importance of non-coding RNAs in pregnancy disorders is also gaining recognition. Munjas et al. [11] reviewed the regulatory role of microRNAs (e.g., miR-210) and long non-coding RNAs in modulating gene expression involved in trophoblast invasion, immune tolerance, and vascular adaptation, further supporting their utility as diagnostic biomarkers for preeclampsia.

From a metabolic standpoint, gestational diabetes mellitus (GDM) has been linked to dysregulated molecular pathways involving adipokines, insulin resistance markers, and inflammatory cytokines [12]. These findings point to a complex interplay between maternal metabolism and placental function that, if disrupted, can affect both maternal and fetal health.

Maternal–fetal communication is another crucial domain. Darbinian et al. [13] investigated exosomal markers in maternal blood, identifying miRNAs and proteins that allow for the early detection of fetal alcohol spectrum disorders, highlighting the potential of liquid biopsy techniques for non-invasive prenatal screening.

The role of immunological and infectious factors in pregnancy outcomes was investigated by Boyle et al. [14], who used murine models to show how perinatal infections disrupt immune gene expression and impair placental vascularization, increasing the risk of miscarriage or fetal growth anomalies.

Alpha-1 antitrypsin has emerged as a regulator of placental angiogenesis, with deficiency potentially contributing to vascular complications such as preeclampsia [15]. This further illustrates how individual protein regulators can have systemic effects on gestational success.

Finally, Martinez-Bueno et al. [16] examined the long-term impact of pregnancy complications like preeclampsia on maternal cardiovascular health, linking persistent epigenetic and endothelial alterations to increased risk of cardiovascular disease, emphasizing the need for postpartum molecular monitoring.

## 5. Final Remarks

The findings presented in these studies enhance our understanding of the molecular underpinnings of pregnancy disorders and suggest potential therapeutic targets for future research. Given the complexity of preeclampsia, prenatal hypoxia, and cervical cancer, multidisciplinary approaches integrating molecular biology, clinical research, and personalized medicine will be crucial for developing effective diagnostic tools and treatment strategies aimed at improving maternal and fetal health.
